# Prevalence and Psychiatric Correlates of Illicit Substance Use in UK Undergraduate Students

**DOI:** 10.3390/brainsci13020360

**Published:** 2023-02-19

**Authors:** Holly Foster, Jodie Stevenson, Umair Akram

**Affiliations:** 1 School of Psychology, University of Lincoln, Lincoln LN17TS, UK; 2 Nuffield Department of Clinical Neurosciences, University of Oxford, Oxford OX1 4BH, UK

**Keywords:** students mental health, drug use, anxiety, depression, insomnia, stress

## Abstract

This study examined the prevalence of illegal drug use in UK students and motivators behind such behavior. Additionally, we explored possible relationships between substance use, psychosocial motivators, and psychiatric distress. A group (*n* = 543) of students completed online measures of substance use, anxiety, depression, perceived stress, and insomnia. A series of reasons behind their use were ranked based on importance. Reported cannabis, cocaine, nitrous oxide, ketamine, and MDMA use were most prevalent based on lifetime, past year, and month assessments. The experience of anxiety, depression, perceived stress, and insomnia were related to increased reports of substance use. Poor self-confidence and self-medication were key motivators of illicit drug use in those presenting greater psychiatric distress. These outcomes add to the sparse body of literature concerning illicit substance use in relation to psychiatric distress amongst UK students. Furthermore, we provided novel insight into the psychosocial motivators of such use.

## 1. Introduction

University level study entails prominent changes in social and academic functioning which may include increased independence from a lack of parental support and oversight and greater pressure to partake in social obligations, both occurring alongside increased academic demands (e.g., independent learning) [1]. Consequently, the university student population remains significantly vulnerable to the development or exacerbation of mental health difficulties [2,3,4]. As a potential coping mechanism [5] or opportunistic experimentation, illicit substance use remains highly prevalent amongst university students in the UK [6]. Lifetime (40%) and yearly (25%) drug use remains high in UK university students [7], even when compared with their non-student young adult counterparts [8]. More recently, of *n* = 7855 UK university students, cannabis emerged as the most prevalent drug used (28%), followed by MDMA (i.e., methylenedioxymethamphetamine; 15%), nitrous oxide (14%), cocaine (5%), and ketamine (4%) [7].

Many studies have examined possible predictors of illicit drug use amongst UK university students [9,10,11,12]. Here, research differentially evidences the experience of poor sleep, stress, anxiety, and depression to be associated with greater reports of illicit drug use, specifically, amongst those studying medicine [13,14], dentistry [15], law [13,16], and the biomedical [17] and social sciences [18]. With that in mind, illicit drug use and the experience of poor mental health often leads to poor academic performance and increased rates of drop-out [19,20].

Whilst previous work explores the prevalence and possible predictors of illicit substance use amongst UK university students, many studies rely on students perusing a specific course. Similar variation emerges in the context of motivational factors, the specific drug type, and psychological correlate examined. As such, this study aimed to further understand the prevalence and psychological factors related to illicit substance use amongst a sample of UK university students. More specifically, the present work aimed to examine (a) the prevalence of substance use in the UK student population; (b) the relationships between substance use, psychosocial determinants, and symptoms of anxiety, depression, perceived stress, and insomnia; and (c) possible psychosocial determinants of illicit drug use in UK university students.

## 2. Materials and Methods

### 2.1. Sample and Procedure

The protocol was approved by the Sheffield Hallam University Research Ethics Committee, and all participants provided online informed consent. A cross-sectional online questionnaire-based study was implemented comprising questions designed to examine the prevalence of illicit drug use and symptoms of anxiety, depression, perceived stress, and insomnia. Undergraduate students from two UK universities were recruited through institutional course credit participation schemes (provided points required for completion of a study module completion), social media (i.e., Facebook, Twitter, Reddit), and faculty emails. Overall, *n* = 600 participants began or accessed the online questionnaire, after incomplete entries were discarded *n* = 543 completed entries (mean age = 20.02 ± 3.16, range 18–48, 82% female, 16.2% male; 1.7% non-binary) were retained for analysis. This sample size was sufficient for a 95% confidence level, exceeding our target of 500 responses leaving an acceptable 4.5% margin of error [21]. SPSS (version 27, IBM Corp, Armond, NY, USA) was used to perform formal statistical analyses, with significance considered at the *p* < 0.05 level. Data were collected during the 2018/19 academic year.

### 2.2. Measures

#### 2.2.1. Substance Use and Motivational Factors

An in-house questionnaire was developed based on the Drug Use Questionnaire for Students [22] and Drug Use Questionnaire for High School Students [23]. Specifically, students indicated (yes/no) whether they have ever consumed the following substances in their lifetime, past twelve months, and the past month: Cannabis (in the UK, cannabis remains an illegal Class B substance); Cocaine; Amphetamines; Methamphetamines; Nootropics; Ketamine; Hallucinogens; MDMA; Nitrous Oxide; Sedative Hypnotics; Heroin; other Opioids. To understand the motivations behind illicit drug use, participants ranked the following reasons from 1 (most important reason) to 10 (least important reason): curiosity; pleasure; provided substance by a friend; peer pressure; low self-confidence; boredom/lack of amusement; using substances as a form of self-medication for physical or psychiatric difficulties; lack of knowledge concerning the risks of substance use; and the presence of addiction in the home. Responses to each substance were summated, yielding composite scores (between 0–10) for lifetime use, past twelve months, and the past month.

#### 2.2.2. Anxiety and Depression

Symptoms of anxiety and depression were assessed using the original version of The Hospital Anxiety and Depression Scale (HADS) [24], consisting of 14 items (seven for both the anxiety and depression subscales) scored between 0 and 3, with a maximum score of 21 on both subscales. Higher scores on each subscale represent greater anxiety and depression. Both subscales demonstrated good internal consistency (Cronbach’s α of 0.86 for anxiety and 0.81 for depression).

#### 2.2.3. Perceived Stress

The Perceived Stress Scale (PSS) examined appraisal of stress levels over the past month [25]. Fourteen items, scored on a 5-point scale (0–4), are summed to provide total scores ranging between 0 and 56. Higher scores indicate higher levels of perceived stress. The internal consistency of the scale in the present study was *α* = 0.86.

#### 2.2.4. Insomnia Symptoms

Insomnia symptoms were assessed using Sleep Condition Indicator (SCI), a clinical screening tool developed to appraise insomnia symptoms against the DSM-5 criteria for Insomnia Disorder [26]. Eight items each scored between 0–4 examine insomnia symptomology during the last month. Specifically, questions pertain to sleep onset latency, awakenings during the night, perceived sleep quality, impairment to daytime functioning, and symptom persistence. Items are summed to create a total score, with lower scores indicating greater insomnia symptom severity. Internal consistency was high in the present sample (α = 0.89).

### 2.3. Statistical Analysis

All analyses were carried out using IBM SPSS v.29.0 (IBM Corp., Armonk, NT, USA). Descriptive presentation of the data includes mean values and standard deviations for each psychometric measure and prevalence of illicit substance use. Next, the percentage prevalence rates for illicit substance use over the lifespan, past twelve months, and past month were calculated. To determine the most cited reasons behind illicit substance use, the mean rank order of the ten motivational reasons were calculated. Here, scored between 1–10, lower rank scores indicated greater motivation behind the specified reason. Next, a series of correlational analyses (Pearson’s bivariate) examined possible relationships between psychiatric symptoms, substance use, and motivational factors. Finally, a series of linear regression analyses were used to assess the most prominent associations between the type of substance used during each assessed period (i.e., predictors) and symptoms of anxiety, depression, perceived stress, and insomnia (i.e., dependent variables). Significance was considered at the *p* < 0.05 level in all analyses.

## 3. Results

### 3.1. Prevalence

Based on yes/no responses, the percentage prevalence rates and composite scores for illicit substance use over the lifespan, past twelve months, and past month are provided in Table 1. Examination of drug type revealed that cannabis use was the most prevalent drug used across all time points (25.8–58.7%). This was followed by the prevalence of cocaine (13.4–31.2%), nitrous oxide (11.2–38.8%), ketamine (13.1–28.6%), and MDMA (11.6–31.2%) use, where similar rates emerged across time points. Opioid (2.6–12.9%), sedative hypnotic (1.7–9.2%), and amphetamine (1.1–8.9%) use followed at a lower rate.

### 3.2. Motivating Factors for Illicit Substance Use

Mean scores and standard deviations for psychological measures and cited reasons for drug use, and the mean rank order for self-reported motivations for engaging in drug use are presented in Table 2. The results demonstrated that most cited reason for substance use was curiosity. In order from highest (i.e., number one reason) to lowest mean rank (*n* = 10), this was followed by pleasure; provided substance by a friend; peer pressure; low self-confidence; boredom/lack of amusement; using substances as a form of self-medication for physical or psychiatric difficulties; lack of knowledge concerning the risks of substance use; and finally, the presence of addiction in the home.

Composite scores for lifetime and substance use over the past year were significantly related to an increased emphasis on boredom, pursuit of pleasure, low cost, and self-medication. Reduced reports of peer pressure, familial influence, self-confidence, and curiosity emerged as protective factors against substance use. Over the past month, substance use was significantly related to increased reports of pleasure and low cost, whereas reduced peer pressure or lack of confidence served as attenuating factors (all *p*’s < 0.05, see Table 3).

### 3.3. Relationships between Psychiatric Symptoms, Illicit Substance Use, and Motivating Factors

A series of Pearson’s correlational analyses explored the relationships between composite substance use scores, symptoms of anxiety, depression, perceived stress and insomnia, and cited reasons for drug use. As expected, composite scores for lifetime, past year, and past month substance use were all significantly associated with greater levels of anxiety, depression, perceived stress, and insomnia symptoms (all *p*’s < 0.05, see Table 3). Increased anxiety was associated with poor self-confidence and an increased tendency to self-medicate, whereas pleasure seeking, low cost, and peer accessibility were related to reduced anxiety. These outcomes were mirrored for depressive symptoms, except for pleasure. Increased levels of perceived stress were associated with poor self-confidence and an increased tendency to self-medicate, whereas reduced stress was related to reports of peer pressure, low cost, and peer accessibility. Like anxiety, depression and stress, the experience of insomnia symptoms was also related to increased reports of low self-confidence and self-medication as cited reasons for substance use (all *p*’s < 0.05, see Table 3).

### 3.4. Substance Type as Predictors of Anxiety, Depression, Stress, and Insomnia

A series of linear regression analysis was used to assess possible associations between the type of substance used during each assessed period (i.e., predictors) and symptoms of anxiety, depression, perceived stress, and insomnia (i.e., dependent variables). Significant predictors of increased anxiety included lifetime use of nootropics, hallucinogens, and opioids (F = 3.73, *p* < 0.001); use of sedatives and opioids during the past year (F = 4.21, *p* < 0.001); and amphetamine and sedative use within the past month (F = 2.90, *p* < 0.001). Next, significant predictors of increased depression included lifetime use of methamphetamines, sedatives, and hallucinogens (F = 4.06, *p* < 0.001); use of amphetamine, sedative, and nitrous oxide during the past year (F = 4.36, *p* < 0.001); and cannabis, amphetamine, and sedative use within the past month (F = 4.35, *p* < 0.001). Further, significant predictors of perceived stress included lifetime use of cannabis, nootropics, and hallucinogens (F = 2.23, *p* < 0.01); use of cannabis, opioids, hallucinogens, and nitrous oxide during the past year (F = 3.19, *p* < 0.001); cannabis and hallucinogens use within the past month (F = 1.93, *p* < 0.05). Finally, significant predictors of insomnia symptoms included lifetime use of cannabis, methamphetamines, nootropics, hallucinogens, and heroin (F = 3.25, *p* < 0.001); use of cannabis and sedatives during the past year (F = 2.67, *p* < 0.001); cannabis, sedatives, and hallucinogens use within the past month (F = 2.62, *p* < 0.01). See Table 4.

## 4. Discussion

The present data revealed a considerable number of undergraduate students reported illicit substance use at least once in their lifetime and during the past 12 months. Over half the sample (58.7%) had used at least one substance in their lifetime. Cannabis use was most used substance across each time point (25.8–58.7%). Similarly, use of cocaine, nitrous oxide, ketamine, and MDMA appear to be equally prevalent. These findings add to the body of evidence examining the prevalence of illicit substance use amongst UK undergraduate university students. The current outcomes are in line with previous studies sampling undergraduate students from England [18] and Wales [10] where cannabis consistently emerges to be the most consumed substance, followed by stimulants. Using an in-house assessment of illicit substance use, Bennet and Holloway [10] found the lifetime (40.2%) and 12-month (21%) prevalence of cannabis use to be comparably high. In contrast, rates of stimulant drug use (cocaine, 14%; ecstasy, 13.8%; amphetamines, 9.1%) remained somewhat lower than those yielded in the present sample. In a more specific sample of dental students in the UK, 55% reported cannabis use at least once or twice following the start of their course, whereas 20.5% reported stimulant use [15]. Whilst most UK studies exploring student substance sample medical students [12,13,16], the pattern of outcomes remains somewhat consistent. Recently, Bogowicz and colleagues [13] found one-quarter of medical and one-third of law students reported the use of illicit substances within the past year, whereby cannabis, cocaine, and extasy, in respective order, were most favored. Here, symptoms of anxiety and depression were significantly related to increased use. Deploying a prospective design, Newbury-Birch and colleagues [16] revealed that 66% of medical and 51% of law students reported using cannabis at least once in their lifetime. More crucially, across each year of study, approximately half of the medical and dental students sampled reported experimenting with illicit drugs. Despite recording the symptoms’ presentation of anxiety, stress, and depression, no longitudinal analysis was employed to explore the possible causal role of psychiatric symptoms as predictors of substance use.

Symptoms of anxiety, depression, perceived stress, and insomnia were related to increased reports of overall substance use in the present study, regardless of the examined timeframe. These results are consistent with existing evidence that psychiatric difficulties are related to increased substance use in the US student population [27]. Our outcomes shed light on the relationship between type(s) of substance used in relation to particular psychiatric symptoms. Here, substance use varied based on the nature of reported difficulties. Apart from perceived stress, sedative use over the past year and month appeared to be related to increased anxiety, depression, and insomnia. In students experiencing such difficulties, the use of non-prescribed sedative hypnotics appears well evidenced in the US, Africa, South Africa, Saudi Arabia, and Turkey [28,29,30,31,32]. Here, students often cite self-medicating with the hope of ameliorating their distress, where in the context of insomnia and anxiety, sedative drugs are considered to reduce the physiological effects of anxiety whilst facilitating sleep onset [33,34]. However, problems with tolerance, particularly with benzodiazepines, may lead to dependence and an increased dose to sustain their effects. The risk of overdose when combined with alcohol remains particularly problematic, where the adverse interaction effects are often unknown to students [35]. In line with previous observations, the current results found cannabis use to be related to increased symptoms of depression, insomnia, and stress [36,37,38]. More specifically, cannabis use over the past year and month was related to increased reports of stress and insomnia, whereas depression was related to use within the month. The experience of negative self-conscious emotions are frequently related to reduced wellbeing and psychiatric disorders including anxiety, depression, insomnia and the self-perception of body image [39,40,41,42]. As such, cannabis use may reflect self-medication as a means of temporarily reduced stress, anxiety, and depressive symptoms, which in turn may improve self-confidence. Likewise, some students may consume cannabis to improve their sleep, and facilitate sleep onset. Certainly, derivatives of cannabis with relaxing and sleep promoting effects can be legally purchased in many countries [43]. These derivatives can increase melatonin production and inhibit wakefulness by activating the cannabinoid type-1 (CB_1_) receptors in the wake-promoting regions of the brain [44,45,46].

Few studies have examined the motivations for student drug use specifically within the UK. Those that have evidence feelings of inadequacy, loneliness, interpersonal difficulties, and peer influence as prominent reasons for engaging with illicit drug use [6,47]. When examining the motivations behind illicit substance use in the present study, we evidenced curiosity and peer pressure, respectively, as the highest ranked reasons for substance use in general. More crucially, poor self-confidence and the notion of self-medication emerged to be the most substantial motivating factors of substance use in those presenting greater psychiatric distress. Whilst poor self-confidence and low self-esteem have been previously related to illicit drug use amongst university students [48,49,50], the present outcomes provide novel insight into the motivational factors which related to the experience of anxiety, depression, perceived stress, and insomnia.

Over the past decade, the experience of psychiatric difficulties appears to be continually rising among university students in the UK, with a five-fold increase in students opting to disclose their mental health difficulties to an institutional support service [51]. Despite this, institutional wellbeing services often fail to meet the increased demand for help and support [52,53]. The experience of psychiatric distress and co-morbid drug use may significantly impair the day to day lives of students in the context of academic, social, and occupational functioning [54]. Accordingly, student wellbeing services and academic institutions in the UK must actively seek to better identify at-risk populations by deploying improved screening processes and systematic monitoring of student mental health and substance use to allow for early intervention [51,55]. Furthermore, new students could be provided information concerning drug safety, dangers of self-medicating, and adverse effects, whilst highlighting alternative avenues based on motivating factors in the form of educational pamphlets and a start of term lecture [2]. Here, for example, in the context of poor self-confidence, the information provided may seek to define and raise awareness concerning the signs of low self-confidence and poor self-esteem whilst signposting students to the relevant internal or external (i.e., NHS Improving Access to Psychological Therapies (IAPT) service) wellbeing resources available. Indeed, students may self-refer to IAPT without the requirement of a general practitioner referral. Information concerning the signs and symptoms of mental health difficulties and the adverse consequences of substance misuse may be deployed in the same manner, or through collaboration with existing mental health organizations in the UK (e.g., University Nightline, Mind UK, The Samaritans).

Several limitations of the present work should be highlighted. The cross-sectional design used here leaves the current outcomes vulnerable to inflation bias between variables and restricts the ability to draw conclusions about causal relationships. In addition, self-reported measures were used for the identification of clinical symptoms, instead of clinical assessments. Moreover, it is possible that students already motivated to take part in a study about illicit drug use completed the survey, possibly indicating a self-selection bias. Whilst alcohol use is legal in the UK for individuals over the age of eighteen, its prevalence was not examined in the current study. Finally, the sample used did not comprise of a heterogeneous population (e.g., white female young adults were overrepresented). With that in mind, research demonstrates that females demonstrate greater willingness to participate in health-related research [56]. Nevertheless, a possible strength includes the use of well-validated scales with robust psychometric properties to examine psychiatric distress, rather than relying on single-item scales or in-house bespoke measures often used with large scale data collection. Further research should take a longitudinal approach amongst a more homogenous sample of university students, possibly tracking student wellbeing and substance use across each year of study, to overcome the current limitations.

To summarize, a large proportion of students reported illicit substance use at least once in their lifetime, and during the past year. The experience of anxiety, depression, perceived stress, and insomnia were related to increased reports of substance use. Poor self-confidence and self-medication were key motivators of illicit drug use in those presenting greater psychiatric distress.

## Figures and Tables

**Table 1:** Percentage frequencies and composite scores (mean ± standard deviation) for lifetime, past year, and past month prevalence of drug use.

Drug Type	Month (*n*)	Year (*n*)	Lifetime (*n*)
Cannabis	25.8% (138)	46.3% (249)	58.7% (316)
Cocaine	13.4% (72)	25.7% (138)	31.2% (168)
Amphetamine	1.1% (6)	4.1% (22)	8.9% (48)
Methamphetamine	0.4% (2)	0.4% (2)	0.6% (3)
Nootropic	1.9% (10)	3.6% (19)	6.3% (34)
Ketamine	13.1% (70)	24.5% (131)	28.6% (154)
Hallucinogenic	3.9% (21)	9.5% (51)	13.3% (72)
MDMA	11.6% (62)	25.2% (135)	31.2% (168)
Nitrous Oxide	11.2% (60)	28.4% (152)	38.8% (209)
Sedative Hypnotics	1.7% (9)	5.6% (30)	9.2% (50)
Heroin	0.4% (2)	0.4% (2)	0.6% (3)
All Other Opioids	2.6% (14)	8.4% (45)	12.9% (70)
Composite Score	0.87 ± 1.52 (466)	1.81 ± 2.69 (976)	2.39 ± 2.63 (1295)

Note: Percentages are based on yes/no responses; n, total number of yes responses.

**Table 2:** Mean scores and standard deviations for psychological measures and cited reasons for drug use.

Measures	Mean ± Standard Deviation
Anxiety	8.88 ± 4.63
Depression	5.50 ± 3.80
Stress	22.27 ± 6.96
Insomnia	17.80 ± 7.90
Cited Reasons for Drug Use ^	
Curiosity	1.84 ± 1.43
Boredom	6.11 ± 1.72
Peer pressure	5.03 ± 2.65
Pleasure	3.12 ± 1.77
Low cost and availability	6.79 ± 2.11
Low self-confidence	5.84 ± 2.06
Self-medication	6.93 ± 2.82
Little knowledge concerning expected and unexpected side effects	6.94 ± 1.77
Parental or sibling addiction	8.86 ± 1.89
Provided by a peer	3.55 ± 1.84

Note: ^ = Scored between 1 and 10, with lower scores indicating a higher mean rank of the cited reason.

**Table 3:** Correlational associations between cited reasons for substance use and symptoms of anxiety, depression, perceived stress, and insomnia.

					Composite Substance Use		
Influencing Factor	Anxiety	Depression	Stress	Insomnia	Past Month	Past Year	Lifetime
Curiosity	0.04	−0.08	−0.01	−0.06	−0.05	−0.03	0.01
Boredom	0.05	0.03	−0.04	−0.07	−0.04	−0.10 *	−0.11 *
Peer pressure	0.08	0.05	0.11 *	0.14	0.21 **	0.29 **	0.29 **
Pleasure	0.10 *	0.09	0.03	−0.06	−0.17 **	−0.27 **	−0.24 **
Low cost/availability	0.12 *	0.16 **	0.15 **	−0.07	−0.17 **	−0.28 **	−0.23 **
Low self-confidence	−0.21 **	−0.13 **	−0.16 **	0.11 *	0.10 *	0.15 **	0.13 **
Self-medication	−0.32 **	−0.21 **	−0.23 **	0.21 **	−0.07	−0.09 *	−0.12 *
Lack of knowledge	0.09	0.05	0.07	−0.06	0.04	0.10 *	0.07
Parental/sibling	0.01	−0.02	0.01	0.01	0.05	0.13 **	0.09
Provided by a peer	0.19 **	0.14 **	0.15 **	−0.05	0.04	0.03	0.04
Composite Substance Use							
Lifetime	0.18 **	0.16 **	0.15 **	−0.11 **	-	-	-
Past Year	0.13 **	0.16 **	0.12 **	−0.13 **	-	-	-
Past Month	0.12 **	0.17 **	0.09 *	−0.11 **	-	-	-

Note: * Sig at < 0.05, ** < 0.01.

**Table 4:** Substance type as predictors of anxiety, depression, stress, and insomnia.

	Month			Year			Lifetime		
	β	*t*	P	β	*t*	P	β	*t*	P
[A] Anxiety	R^2^ = 0.06			R^2^ = 0.08			R^2^ = 0.08		
Cannabis	0.434	0.841	0.401	0.399	0.842	0.400	0.846	0.090	0.074
Cocaine	−0.196	−0.267	0.790	−0.129	−0.199	0.843	0.458	0.046	0.496
Amphetamine	6.914	2.796	0.005 **	2.028	1.817	0.070	1.397	0.085	0.112
Methamphetamine	-	-	-	-	-	-	6.684	0.108	0.062
Nootropic	−2.014	−1.180	0.239	0.556	0.474	0.635	2.046	0.108	0.025 *
Ketamine	0.655	0.829	0.407	−0.212	−0.311	0.756	−0.059	−0.006	0.925
Hallucinogenic	−1.722	−1.499	0.135	−1.513	−1.846	0.066	−1.947	−0.143	0.013 *
MDMA	−0.397	−0.518	0.605	0.435	0.635	0.526	0.384	0.038	0.560
Nitrous Oxide	0.292	0.393	0.695	−0.738	−1.330	0.184	−0.891	−0.094	0.102
Sedative Hypnotics	5.432	3.046	0.002 **	2.030	2.066	0.039 *	1.354	0.084	0.104
Heroin	0.613	0.150	0.881	5.069	1.489	0.137	−3.311	−0.053	0.357
All Other Opioids	0.813	0.607	0.544	3.287	4.150	0.000 **	1.704	0.122	0.012 *
[B] Depression	R^2^ = 0.08			R^2^ = 0.08			R^2^ = 0.09		
Cannabis	0.883	2.116	0.035 *	−0.085	−0.219	0.827	0.419	1.087	0.278
Cocaine	−0.117	−0.197	0.844	−0.001	−0.001	0.999	0.425	0.774	0.439
Amphetamine	5.799	2.901	0.004 **	2.074	2.268	0.024 *	1.202	1.680	0.094
Methamphetamine	-	-	-	-	-	-	6.569	2.254	0.025 *
Nootropic	−1.295	−0.939	0.348	1.040	1.082	0.280	1.436	1.940	0.053
Ketamine	−0.289	−0.452	0.651	0.703	1.258	0.209	0.336	0.653	0.514
Hallucinogenic	−1.635	−1.761	0.079	−1.052	−1.566	0.118	−1.715	−2.679	0.008 **
MDMA	0.576	0.928	0.354	0.442	0.786	0.432	0.566	1.054	0.292
Nitrous Oxide	0.186	0.311	0.756	−0.839	−1.845	0.066	−1.160	−2.615	0.009 **
Sedative Hypnotics	3.545	2.459	0.014 *	1.726	2.143	0.033 *	1.351	1.994	0.047 *
Heroin	5.869	1.781	0.076	9.375	3.360	0.001 **	1.151	0.393	0.695
All Other Opioids	0.419	0.387	0.699	0.801	1.234	0.218	0.471	0.857	0.392
[C] Stress	R^2^ = 0.04			R^2^ = 0.06			R^2^ = 0.05		
Cannabis	1.669	2.124	0.034 *	1.618	2.238	0.026 *	1.551	2.151	0.032 *
Cocaine	−0.748	−0.668	0.504	0.134	0.135	0.892	0.693	0.675	0.500
Amphetamine	5.622	1.494	0.136	2.449	1.441	0.150	1.217	0.910	0.363
Methamphetamine	-	-	-	-	-	-	0.828	0.152	0.879
Nootropic	1.126	0.434	0.665	2.730	1.528	0.127	3.404	2.461	0.014 *
Ketamine	−0.759	−0.632	0.528	−0.579	−0.558	0.577	0.757	0.787	0.431
Hallucinogenic	−3.805	−2.177	0.030 *	−3.194	−2.557	0.011 *	−3.018	−2.522	0.012 *
MDMA	0.075	0.064	0.949	1.198	1.146	0.252	0.259	0.258	0.796
Nitrous Oxide	1.230	1.089	0.277	−1.675	−1.982	0.048 *	−1.024	−1.235	0.217
Sedative Hypnotics	4.899	1.806	0.072	1.651	1.103	0.271	1.459	1.152	0.250
Heroin	−0.342	−0.055	0.956	2.106	0.406	0.685	−0.824	−0.150	0.881
All Other Opioids	1.084	0.532	0.595	3.471	2.877	0.004 **	0.765	0.744	0.457
[D] Insomnia	R^2^ = 0.05			R^2^ = 0.05			R^2^ = 0.07		
Cannabis	−2.065	−2.339	0.020 *	−1.872	−2.269	0.024 *	−1.641	−2.027	0.043 *
Cocaine	0.064	0.051	0.960	0.178	0.157	0.875	−1.321	−1.146	0.252
Amphetamine	−8.738	−2.067	0.039 *	−2.513	−1.296	0.196	−2.587	−1.723	0.085
Methamphetamine	-	-	-	-	-	-	−20.231	−3.307	0.001 **
Nootropic	4.621	1.584	0.114	−0.749	−0.368	0.713	−3.771	−2.428	0.016 **
Ketamine	−0.926	−0.686	0.493	−0.418	−0.353	0.724	−0.109	−0.101	0.920
Hallucinogenic	4.366	2.223	0.027 *	2.559	1.796	0.073	3.751	2.792	0.005 **
MDMA	−0.286	−0.218	0.828	−0.575	−0.483	0.630	−0.876	−0.777	0.438
Nitrous Oxide	0.485	0.382	0.703	1.643	1.704	0.089	2.556	2.746	0.006 **
Sedative Hypnotics	−8.929	−2.929	0.004 **	−3.938	−2.306	0.022 *	−1.077	−0.758	0.449
Heroin	−1.241	−0.178	0.859	−6.010	−1.016	0.310	14.332	2.330	0.020 *
All Other Opioids	−0.791	−0.346	0.730	−2.144	−1.558	0.120	−1.159	−1.004	0.316

Note: * Sig at < 0.05, ** < 0.01.

## Data Availability

Data will be made available on reasonable request.
